# Designing agricultural grasses to help mitigate proteolysis during ensiling to optimize protein feed provisions for livestock

**DOI:** 10.1002/fes3.475

**Published:** 2023-06-13

**Authors:** Nuwan P. K. Muhandiram, Michael W. Humphreys, Rhun Fychan, John W. Davies, Ruth Sanderson, Christina L. Marley

**Affiliations:** ^1^ Institute of Biological, Environmental and Rural Sciences (IBERS) Aberystwyth University Aberystwyth UK

**Keywords:** *Festulolium*, forage conservation, protein, ryegrass

## Abstract

The efficient preservation of protein in silage for livestock feed is dependent on the rate and extent of proteolysis. Previous research on fresh forage indicated enhanced protein stability in certain *Festulolium* (ryegrass *×* fescue hybrids) cultivars compared to ryegrass. This is the first report of an experiment to test the hypothesis that a *Lolium perenne × Festuca arundinacea var glaucescens* cultivar had reduced proteolysis compared to perennial ryegrass (*L. perenne*) during the ensiling process. Forages were harvested in May (Cut 2) and August (Cut 4), wilted for 24 h and ensiled in laboratory‐scale silos. Silage was destructively sampled at 0 h, 9 h, 24 h, 48 h, 72 h, 14 days and 90 days post‐ensiling, and dry matter (DM), pH and chemical composition were determined. At Cut 2, there was no difference in crude protein between treatments but ryegrass had higher soluble nitrogen (SN) (*P* < 0.001) and grass × time interactions (*p* = 0.03) indicated higher rates of proteolysis. By Cut 4, *Festulolium* had (5.5% units) higher CP than ryegrass (*p* < 0.001) but SN did not differ. Ammonia‐N did not differ between silages in either cut. DM differences (11.8% units) between treatments in Cut 4 (v.2.2% in Cut 2) may have masked effects on proteolysis, highlighting the importance of management on silage quality. This was despite higher WSC in ryegrass in both cuts (*p <* 0.001), with grass × time interactions (Cut 2; *p* = 0.03) showing slower WSC decline in ryegrass in Cut 4 (*p* < 0.001). Silage pH values did not differ between grasses in either cut, but grass × time interactions (*p* < 0.001) showed a slower decline in both ryegrass cuts, resulting in higher (*p* < 0.05) pH at 24 h and 72 h for Cuts 2 and 4, respectively. Overall, the hypothesis for an enhanced protein stability in *Festulolium* when ensiled as ruminant feed was evidenced by lower SN but not ammonia‐N in an early‐cut silage with a comparable DM to ryegrass.

## Abstract 2

An experiment tested the hypothesis that a Festulolium had reduced proteolysis compared to perennial ryegrass when ensiled. The benefits of Festulolium silage as ruminant feed were evidenced by lower soluble nitrogen but not ammonia‐N in an early‐cut silage with a comparable DM to ryegrass.

## 1 INTRODUCTION

Grasslands cover 25% of the Earth's land surface area, equating to approximately 3.5 billion hectares (Lemaire et al., 2011), and 70% of the global agricultural land area (Reynolds & Frame, 2005). Through sustainable practices and management, grasslands provide efficient, healthy and cost‐effective supplies of ruminant livestock feed, whilst also delivering environmental services (Humphreys, O’Donovan, & Sheehy‐Skeffington, 2014; Lemaire et al., 2011). Livestock provides one third of the protein consumed in human diets globally and their products are rich in protein and essential nutrients recognized for their role in global food and nutrition security (Smith et al., 2013).

Silage is the main winter‐conserved forage option for livestock systems in the UK and in other European countries such as Germany, Denmark and the Netherlands (Mayne, 1993; Wilkinson et al., 1996). Loss of N from ruminant livestock poses a significant environmental risk due to nitrous oxide and methane emissions from excreta (Cardenas et al., 2007) and silage effluent (Green, 2019). Conversely, if these N excretions are captured and effectively stored, they can significantly reduce reliance on inorganic N fertilizers and boost productivity, when animal manures are applied to grassland soils (Crotty et al., 2014). Advances in silage technology and plant breeding through the development of more sustainable crop cultivars can help mitigate some of the potential environmental impacts from livestock agriculture. Grass varieties have been developed to help provide resilience to the contrasting and more extreme abiotic stresses increasingly experienced due to climate change, providing new tools for farmers to help safeguard forage supply and increase feed efficiency in grassland‐based agri‐food systems. *Festulolium* cultivars are grasses generated through conventional plant hybridization technologies that can combine the complementary agronomic attributes found in ryegrasses (*Lolium* spp.) and the stress resilience of related fescue (*Festuca* spp.) species (Humphreys & Zwierzykowski, 2020) during different environmental conditions (Loka et al., 2019).

Improved protein feed security may be achieved through greater nitrogen use efficiency (NUE) in ruminant livestock systems (Marley, Fraser, et al., 2007; Marley, Fychan, et al., 2007) and, in turn, NUE in ruminants is dependent on the rate of conversion of plant nitrogen (N) to animal protein (Ulyatt et al., 1988). One way to improve ruminant NUE is to reduce the rapid proteolysis of plant protein catalysed initially by endogenous plant, and subsequently microbial, proteases following ingestion by livestock (Kingston‐Smith et al., 2005; Wallace, 1996). Another approach, which is the focus here, is to reduce plant proteolysis that occurs during the ensiling and storage of conserved forage, particularly during the early stages of ensiling if the fermentation rate is too slow to ensure a rapid pH decline (Charmley & Veira, 1991; Tremblay et al., 2011; Winters et al., 2000). Proteolysis of forage in the rumen or *in silo* not only leads to losses of protein in silage effluent (Borreani et al., 2018) prior to use as livestock feed but also reduces the protein available for digestion and amino acids absorbed post‐ruminally (Min et al., 2003). This is because the amino acid‐N in the silage is converted to ammonia, which is readily absorbed across the rumen wall and excreted as a waste product after hepatic conversion into urea (Beever, 1993; MacRae & Ulyatt, 1974). Proteolysis can further create an increased environmental risk due to the high biological oxygen demand of silage effluent on reaching watercourses (Wilkins, 2019).

To ensure appropriate protein quality in feed for livestock, various studies have been conducted with the aim of reducing proteolysis during ensilage. These include optimizing content of dry matter (Muck, 1988), use of additives (Muck et al., 2018; Winters et al., 2001), water‐soluble carbohydrate (Davies et al., 2010) and acids, heat or protein complexes in silage forage (Salawu et al., 1999). McDonald et al. (1991) showed that rapid pH reduction during ensiling reduced the extent of protein breakdown. Secondary plant compounds in forages, such as tannins in birdsfoot trefoil (*Lotus corniculatus*) (Marley et al., 2006) and polyphenol oxidases in red clover (*Trifolium pratense*) (Jones et al., 1995; Marley et al., 2003) also reduce proteolysis during ensiling (Niderkorn & Jayanegara, 2021).

In *Festulolium*, Shaw (2006) found that the rate of protein breakdown in *L. perenne* and *L. multiflorum* was approximately four times faster than the rate observed in *F. arundinacea var. glaucescens* when exposed to in vitro rumen‐like conditions. Humphreys, O’Donovan, Farrell, et al. (2014) demonstrated that expression of the *Festuca*‐based trait was retained in the *Festulolium* amphiploid hybrids *L. perenne* × *F. arundinacea var. glaucescens* (2*n* = 4x = 28) and *L. multiflorum × F. arundinacea var. glaucescens* (2*n* = 4x = 28) when the *Lolium* and *Festuca* parental genome compositions were in balance. These studies were conducted in an in vitro system designed to assess only the plant‐mediated proteolysis potential.

To understand the potential of the *Festulolium* hybrids to decrease the environmental impact in a pasture‐based ruminant system, Kamau et al. (2020) used a semi‐continuous flow fermentation system to simulate rumen‐like conditions and showed that an amphiploid *Festulolium* cultivar of *L. multiflorum × F. arundinacea var. glaucescens* and another *Festulolium* hybrid involving as parents *L. perenne* and the thermotolerant North African fescue *F. mairei* demonstrated significantly lower plant‐mediated proteolysis than the *Lolium* control. However, there have been no studies to determine if these forages have reduced proteolysis in comparison with ryegrass during the ensiling process which, in turn, has an impact on the protein quality of that silage as ruminant feed and ultimately protein feed security.

The aim of this study was to test the hypothesis that the rates and extent of protein breakdown of *Festulolium* cultivar, *L. perenne* × *F. arundinacea var glaucescens*, previously shown to have an enhanced protein stability due to heat‐shock proteins present in fresh forage, were lower when this forage was fermented and conserved as silage when compared to a *L. perenne* control. The research approach was to determine the breakdown of forage protein into soluble nitrogen and ammonia nitrogen to provide outcomes that were relevant to and of interest to end‐users to enable the research findings to be communicated effectively to the industry.

## 2 MATERIALS AND METHODS

### 2.1 Experimental forages and site management

The experimental design comprised two forage treatments: an amphiploid intermediate‐heading *Festulolium* hybrid (*Lolium perenne × Festuca arundinacea var glaucescens*) (referred to as Lp × Fg). The cultivar origins, field performance and potential as efficient freshly harvested livestock feed were referenced in Humphreys, O’Donovan, Farrell, et al. (2014) and its root ontogeny in Humphreys et al. (2018). The *Festulolium* cultivar was compared to an intermediate‐heading perennial ryegrass (*Lolium perenne*) cv AberMagic (referred to as Lp) as control. AberMagic is known as a “high‐sugar” perennial ryegrass because of its higher water‐soluble carbohydrate (WSC) (Wilkins & Humphreys, 2003), which is regularly used commercially in grasslands globally. Samples of both grasses, freshly harvested, were previously used by Kamau et al. (2020) in a comparative study of plant proteolysis under simulated rumen‐like conditions.

Monoculture 3 × 3 m field plots of each treatment were established in August 2014 in four replicate blocks in a randomized design at the Institute of Biological, Environmental and Rural Sciences (IBERS), Wales, Aberystwyth, (52° 26′ 9′′N, 4° 1′ 43′′ W, 20 m of altitude) on soil of the Denbigh series, a well‐drained, fine loamy and silty over rock. Grass plots received 240 kg N/ha/annum, applied in four equal doses, following Cut 1 to Cut 4 of a 5‐weekly silage cutting regime.

### 2.2 Experimental design and silage treatments

Experimental forage was harvested at the height of 5 cm using a Haldrup 1500 plot harvester (J. Haldrup a/s, Løgstør, Denmark) on 31st May 2016 (Cut 2) and 9th August 2016 (Cut 4). Harvested forage from four replicate plots of each treatment were bulked and sub‐sampled. Broad‐leaved weeds and grass weeds were manually removed from each sub‐sample prior to ensiling. Forage samples (circa 14 and 13 kg per treatment at cuts 2 and 4 respectively) were wilted for 24 h (turned 7 h post‐cutting and moved for indoor wilting on a black polythene sheet in a well‐ventilated shed). Both grasses were treated with a *Lactobacillus plantarum* silage inoculant (Wynnstay Dominator, Wynnstay Group Plc, Powys, UK) at the manufacturers recommended rate (1.1 kg dissolved in 200‐L water for 100 t forage). Forage was chopped using a forage harvester (Lely Storm 130P, Netherlands) to a target average chop length of 30 mm. In the laboratory, representative triplicate 100‐g sub‐samples of the two wilted forages were packed by hand into 160‐mL sterile glass tube laboratory silos. Tubes were packed to a standard density, sealed with airlocks containing liquid paraffin and stored in the dark at a constant 21°C. Three replicate silos of each grass treatment were opened and destructively sampled at seven time points: 0 h, 9 h, 1 d, 2 d, 3 d, 14 d and 90 days post‐ensiling respectively.

### 2.3 Silage dry matter and chemical composition

All samples were stored at −20°C prior to subsequent chemical analysis. The DM content of the fresh wilted forages and the silage samples was determined by freeze‐drying. Total nitrogen (TN) concentrations were determined using a Leco FP 428 nitrogen analyser (Leco Corporation, St. Joseph, MI, US). Ammonia‐N (NH_3_‐N) concentrations were determined enzymatically using glutamate dehydrogenase on a discrete analyser (FP‐901M Chemistry Analyzer, Labsystems Oy, Helsinki, Finland; Test kit No. 66–50, Sigma‐Aldrich Co. Ltd., Poole, Dorset, UK). and WSC concentrations by the method of Thomas (1977). Soluble nitrogen content was determined by incubating dried and ground silage samples in filter bags (F57; ANKOM Technology, Macedon, NY, USA) in borate–phosphate buffer (NorFor, 2013) at 39°C for 1 h. Total nitrogen content in the dried sample residues was determined as above. Silage pH was determined as described by Cussen et al. (1995).

### 2.4 Statistical analysis

To examine the effects of grasses and ensiling times and their interactions, analysis of variance was carried out within each harvest date on the 2 × 7 factorial design using GenStat® (Release 21; Baird et al., 2020).

## 3 RESULTS AND DISCUSSION

### 3.1 Fresh forage

The DM content, WSC, total N and soluble N concentrations in the freshly harvested forages are presented in Table 1. The perennial ryegrass cv AberMagic, used as a control, is an example of a “high‐sugar” (i.e., having high WSC) perennial ryegrass cultivar developed at IBERS over the last 40 years (Wilkins & Humphreys, 2003). High sugar ryegrasses help mitigate the rumen imbalance in energy availability and utilization in ruminants by ensuring there is sufficient energy to avoid rumen microbes deaminating amino acids and using their carbon skeletons as an energy source, which otherwise results in the loss of amino‐acid‐N as ammonia and urea (Beever, 1993). The use of such grasses improves protein use efficiency in ruminants (Marley, Fraser, et al., 2007) helping to redress some of the excess N losses and harmful greenhouse gas emissions from livestock agriculture (Wilkins & Humphreys, 2003). The *L. perenne* parent of the amphiploid *Festulolium* cv Lp × Fg cultivar used in the current study was also a “high‐sugar” ryegrass. Humphreys and Zwierzykowski (2020) reported that several forage quality and growth traits expressed in amphiploid *Festulolium* cultivars developed at IBERS involving hybrids of high sugar ryegrasses species when in combination with *F. arundinacea var glaucescens* (or *F. mairei*) would not differ statistically from, and would be determined by, their ryegrass parent.

**TABLE 1:** Mean DM and chemical composition of two grass cultivars, perennial ryegrass (*L. perenne* L.) cv AberMagic; and *Festulolium cv* Lp × Fg (*L. perenne L.* × *F. arundinacea var glaucescens* Roth.) prior to wilting (fresh forage) for use in the silage experiment.

		Grass		s.e.m.	Prob
		Ryegrass	Lp × Fg		
Cut 2	FD DM (g/Kg)	203.3	177.9	0.89	<0.001
	WSC (g/Kg FD DM)	161.9	162.9	4.20	0.882
	Total N (g/Kg FD DM)	24.9	26.2	0.61	0.213
Cut 4	FD DM (g/Kg)	174.2	173.7	17.34	0.983
	WSC (g/Kg FD DM)	151.7	144.1	12.49	0.686
	Total N (g/Kg FD DM)	31.4	32.1	1.53	0.763

Ryegrass (Lp) had a higher DM content than Lp × Fg in the fresh forage at Cut 2 (*p* < 0.001) but DM content did not differ between the two grasses at Cut 4. Data from the larger main plot experiment, from which the forages used in the current study were derived, showed that the yield of the perennial ryegrass was higher than the *Festulolium* in Cut 2 (3.04 v 3.61 t DM/ha), whereas the opposite was found later in the season at Cut 4 (3.26 v. 2.64 t DM/ha). This was despite the experimental plots being cut at regular intervals to maintain the forages in a similar vegetative state. This would imply that the two forages have different growth rates and ontogeny at the two harvest dates. An explanation was their differing ploidy and their species' genome compositions, AberMagic being a diploid perennial ryegrass and Lp × Fg an allotetraploid ryegrass × fescue species' hybrid with double the Lp genome composition. It is also worth noting that sub‐samples of this fresh material were taken for the time course lab‐silos which, given the silo size means that fresh and ensiled material should not be considered a measure of the same sample from a time course perspective. There were no other significant differences between the two grasses in their quality traits measured in Cuts 2 and 4. This is in agreement with previous studies involving the two cultivars, Lp cv AberMagic and Lp × Fg (Collins et al., 2019; Ghesquière et al., 2016; Humphreys, O’Donovan, Farrell, et al., 2014; Kamau et al., 2020).

### 3.2 Silage

Overall, the hypothesis that the rates and extent of protein breakdown of *Festulolium* cultivar, *L. perenne* × *F. arundinacea var glaucescens*, previously shown to have an enhanced protein stability due to heat‐shock proteins present in fresh forage, when ensiled as ruminant feed was evidenced by lower SN but not ammonia‐N in an early‐cut silage with a comparable DM to ryegrass.

### 3.3 Nitrogen and proteolysis

In addition to having comparable forage production and quality to perennial ryegrass (Humphreys, O’Donovan, & Sheehy‐Skeffington, 2014), the *Festulolium* Lp × Fg, as freshly harvested forage feed, has demonstrated potential additional benefits in terms of its greater protein provisions as livestock feed (e.g., Kamau et al., 2020). As described in Humphreys, O’Donovan, Farrell, et al. (2014), the benefits derived from a capability in *Lolium* spp. × *F. arundinacea var glaucescens* (4x) (and in *Lolium* spp. × *F. mairei* (4x) Kamau et al., 2020), hybrids to mitigate plant‐mediated proteolysis. This is frequently initiated in freshly ingested living plant material once eaten and following encounter with the stress conditions found within the rumen. These rumen stresses include a heat shock of 39°C and anoxic conditions, which typically initiate a stress response in living plant cells regularly initiating plant‐mediated proteolysis and release of N‐based compounds which culminate in harmful greenhouse gas emissions into the environment (Kingston‐Smith et al., 2006). Shaw (2006) suggested protective heat‐shock proteins known to be active in certain fescue species indigenous to hot dry Mediterranean climates, e.g., *F. arundinacea var glaucescens*, may confer an equivalent protection to mitigate plant protein degradation in the rumen. Shaw (2006) demonstrated that heat‐shock proteins, active in *F. arundinacea var glaucescens*, are for several hours inactive in ryegrasses and therefore at that time incapable of providing equivalent benefits in protein protection, to that offered by the fescue. Shaw (2006) determined that plant‐mediated proteolysis by perennial ryegrass in rumen‐simulated conditions could amount to four times that found in *F. arundinacea var glaucescens*. An alternative mechanism to limit protein catabolism in *Festulolium* hybrids with slower plant‐mediated proteolysis has also been suggested (Kamau et al., 2018). This involves the partitioning of amino acid catabolism towards branched‐chain amino acids and microbial protein synthesis in relevant *Festuca* species and *Festulolium*, in comparison with ryegrass.

Whilst these reports in totality provide some promise and support for the incorporation of relevant *Festulolium* cultivars as aides to enhance protein retention from freshly grazed grassland, there has been prior to the current study, no equivalent assessment as to whether potential similar benefits in protein retention extend to cut, chopped and ensiled forage of *Festulolium*, as opposed to equivalent conserved ryegrass. An advantage in TN (and hence Crude Protein (CP), which is calculated from those TN values) retention in *Festulolium* silage was indeed observed in the current study, but not consistently at both harvests. The *Festulolium* Lp × Fg silage was found to have higher TN than the ryegrass at Cut 4 (*p* < 0.001), but not at Cut 2 (*p* = 0.092). Unfortunately, no difference in ammonia‐N content was evident in the ensiled grasses neither at Cut 2 nor at Cut 4, which was contrary to the suggestions described by Kamau et al. (2018) for freshly grazed grasses. Ammonia release increased over time of ensiling by the two grasses, both in Cuts 2 and 4 (*p* < 0.001).

Soluble N (SN) comprises short peptide, ammonia and free amino acid production (Winters et al., 2000). In the current study, at Cut 2, ryegrass (Lp) silage had at all time points a higher SN than Lp × Fg (*p* < 0.001), indicative of greater proteolysis by the ryegrass. However, only at the initial post‐wilting stage (Time 0 in Cut 2), and prior to ensiling, were the grasses significantly different. The Lp × Fg had significantly lower SN than was found in the ryegrass samples, indicating that mechanisms, when compared to those in ryegrass, were active in the *Festulolium* and helped to reduce N release prior to ensiling (Table 2). An interaction of grass type and ensiling time was found to be significant (*p* = 0.03) in grasses harvested from Cut 2 relevant to this initial stage. This early advantage and lower SN found in the *Festulolium* persisted subsequently throughout the 90 days of ensiling for all samples taken from Cut 2. This indicates a more rapid protein breakdown in the ryegrass compared to the *Festulolium* cultivar in an early‐season silage. However, at Cut 4, there were no differences found in SN between the grass cultivars in any silage treatment (*p* = 0.99). As with Cut 2, the SN content of silage increased (*p* < 0.001) with ensiling time regardless of grass type or harvest cut, indicative of ongoing proteolytic activities (Der Bedrosian et al., 2012). There were no significant interactions of grass and ensiling time from Cut 4 (*p =* 0.8).

**TABLE 2:** Mean DM, pH and chemical composition of silage prepared from two grass cultivars (G), perennial ryegrass (*L. perenne* L.) cv AberMagic; and *Festulolium cv* Lp × Fg (*L. perenne L.* × *F. arundinacea var glaucescens* Roth.) at the second harvest cut and destructively sampled over six ensiling time points (ET).

Cut 2	Grass (G)	Ensiling time (ET)							Mean	Effect	s.e.m	Prob
		0 h	9 h	1 d	2 d	3 d	14 d	90 d				
FD‐DM g/Kg	Ryegrass	346.1	355.4	357.8	360.0	353.1	346.8	347.3	^A^352.3	G	1.14	<0.001
	Lp × Fg	327.6	332.2	333.4	331.7	331.3	326.6	327.3	^B^330.0	ET	2.14	0.007
	Mean	336.8^b^	343.8^ab^	345.6^a^	345.8^ab^	342.2^ab^	336.7^b^	337.3^ab^		G × ET	3.03	0.732
pH	Ryegrass	6.00^a^	^A^6.03^a^	^A^4.88^b^	4.01^c^	3.80^d^	3.54^e^	3.58^e^	4.55	G	0.005	<0.001
	Lp × Fg	6.04^a^	^B^5.82^b^	^B^4.61^c^	4.04^d^	3.82^e^	3.57^f^	3.62^f^	4.50	ET	0.009	<0.001
	Mean	6.02	5.93	4.74	4.02	3.81	3.56	3.60		G × ET	0.013	<0.001
WSC g/Kg FD DM	Ryegrass	144.8^a^	124.7^b^	104.1^c^	81.0^d^	76.4^d^	49.6^e^	37.7^e^	88.8	G	1.22	<0.001
	Lp × Fg	130.9^a^	128.1^b^	96.8^c^	71.6^d^	66.8^e^	32.9^f^	33.1^f^	80.3	ET	2.29	<0.001
	Mean	137.8	127.0	100.2	76.8	72.2	41.4	36.5		G × ET	3.24	0.027
TN g/Kg FD DM	Ryegrass	26.3	28.2	27.8	27.2	26.5	27.8	28.3	27.4	G	0.14	0.092
	Lp × Fg	26.5	28.0	27.6	27.8	27.6	28.1	28.8	27.8	ET	0.25	<0.001
	Mean	26.4^d^	28.1^ab^	27.7^abc^	27.5^bc^	27.1^cd^	28.0^abc^	28.6^a^		G × ET	0.36	0.540
Soluble N g/Kg TN	Ryegrass	^A^532^a^	512^a^	^A^569^ab^	603^ab^	637^abc^	590^bc^	640^bc^	583	G	5.2	<0.001
	Lp × Fg	^B^453^a^	474^a^	^B^497^a^	576^a^	603^b^	611^c^	605^c^	545	ET	9.8	<0.001
	Mean	493	493	533	589	620	600	623		G × ET	13.8	0.028
Ammonia‐N g/Kg TN	Ryegrass	33.8	46.8	59.3	60.9	44.8	48.7	77.2	53.3	G	1.87	0.869
	Lp × Fg	32.9	41.2	53.3	55.4	46.2	53.1	84.3	52.8	ET	3.5	<0.001
	Mean	33.4^c^	44.2^bcd^	56.1^b^	57.9^b^	45.4^bc^	51.0^b^	83.4^a^		G × ET	4.95	0.726

*Note*: Differing superscripts within rows denote statistically different means based on a Student–Newman–Keuls test (*p* < 0.05).

### 3.4 Other factors affecting proteolysis during ensilage

#### 3.4.1 Wilting and dry matter content

Muck (1988) reported that the extent of ammonia and non‐protein N production in silage was affected by DM content. Freshly harvested forage prior to wilting and ensiling of both the ryegrass and the *Festulolium* at both harvest times were equivalent for WSC, TN and SN content. However, their DM at Cut 2 differed with the ryegrass having greater DM content. This was no longer the case at Cut 4 where there was no significant difference between the grasses in their DM (Table 1). Subsequent to wilting, and consistently over both harvest cuts, the ryegrass‐based silage (Lp) had higher DM when compared to the *Festulolium* cultivar Lp × Fg (*p* < 0.001) (Tables 2 and 3). In the early season (Cut 2), the DM content of the two freshly cut grasses differed (25 g/Kg (Lp) compared to 22 g/Kg (Lp × Fg)) after the 24‐h wilt. In contrast, the later season (Cut 4) forage samples, although having had similar DM values prior to wilting, later had DM content following 24‐h wilting, where Lp was 119 g/Kg higher than the DM content of Lp × Fg. It is well established that DM content of silage will alter the extent of ammonia and non‐protein N production (Muck, 1988) amongst other silage traits. Therefore, the DM difference in Cut 4 silages needs consideration when comparing these results to other findings. That said, it is common practice, and has been widely reported that plant materials having different DM values are often compared in silage studies (e.g., Dewhurst et al., 2003; Fitzgerald & Murphy, 1999; Marley, Fraser, et al., 2007; Marley, Fychan, et al., 2007). DM content is just one of the factors that might influence plant proteolysis during harvesting and conservation, including plant‐mediated endogenous plant enzymes being released (Kingston‐Smith et al., 2005).

**TABLE 3:** Mean DM, pH and chemical composition of silage prepared from two grass cultivars (G), perennial ryegrass (*L. perenne* L.) cv AberMagic; and *Festulolium cv* Lp × Fg (*L. perenne L.* × *F. arundinacea var glaucescens* Roth.) at the fourth harvest cut and destructively sampled over six ensiling time points (ET).

Cut 4	Grass (G)	Ensiling time (ET)							Mean	Effect	s.e.m.	Prob
		0 h	9 h	1 d	2 d	3 d	14 d	90 d				
FD‐DM g/Kg	Ryegrass	438.4	442.1	440.8	443.7	448.2	448.9	429.4	441.6^A^	G	1.85	<0.001
	Lp × Fg	319.3	327.0	330.5	315.8	329.5	327.9	314.2	323.5^B^	ET	3.47	0.018
	Mean	378.8^ab^	384.5^ab^	385.6^ab^	379.8^ab^	388.8^a^	388.4^a^	371.8^b^		G × ET	4.90	0.696
pH	Ryegrass	6.14^a^	6.16^a^	^A^6.11^a^	^A^5.17^b^	^A^4.49^c^	3.84^d^	3.97^d^	5.12	G	0.015	<0.001
	Lp × Fg	6.21^a^	6.28^a^	^B^5.30^b^	^B^4.28^c^	^B^4.04^d^	3.78^e^	3.93^e^	4.83	ET	0.029	<0.001
	Mean	6.18	6.22	5.71	4.72	4.26	3.81	3.95		G × ET	0.041	<0.001
WSC g/Kg FD DM	Ryegrass	^A^142.1^a^	^A^133.1^ab^	^A^122.9^b^	^A^108.8^c^	^A^100.4^c^	^A^71.8^d^	^A^68.9^d^	106.9	G	0.92	<0.001
	Lp × Fg	^B^126.9^a^	^B^106.6^b^	^B^90.3^c^	^B^55.1^d^	^B^48.2^e^	^B^24.6^f^	^B^22.2^f^	67.7	ET	1.73	<0.001
	Mean	134.5	119.8	106.6	81.9	74.3	48.2	45.6		G × ET	2.45	<0.001
TN g/Kg FD DM	Ryegrass	30.6	30.6	31.2	31.0	30.8	30.6	30.8	30.8^B^	G	0.13	<0.001
	Lp × Fg	31.2	32.1	32.2	32.0	32.0	32.4	32.2	32.0^A^	ET	0.24	0.451
	Mean	30.9	31.4	31.7	31.5	31.4	31.5	31.5		G × ET	0.34	0.677
Soluble N g/Kg TN	Ryegrass	350	410	463	521	548	604	650	507	G	6.2	0.987
	Lp × Fg	360	409	490	503	528	597	662	507	ET	11.6	<0.001
	Mean	355^f^	409^e^	477^d^	512^c^	538^c^	600^b^	656^a^		G × ET	16.4	0.782
Ammonia‐N g/Kg TN	Ryegrass	17.6	15.9	22.6	35.8	40.1	55.5	63.5	35.9	G	1.76	0.545
	Lp × Fg	14.1	18.9	23.3	43.6	36.7	52.2	73.0	37.4	ET	3.28	<0.001
	Mean	15.9^d^	17.4^d^	23.0^d^	39.7^c^	38.4^c^	53.8^b^	68.3^a^		G × ET	4.64	0.660

*Note*: Differing superscripts within rows denote statistically different means based on a Student–Newman–Keuls test (*p* < 0.05).

Weather conditions prior to harvest and particularly during wilting will have an impact on the efficiency of ensilage by its influence on the DM achieved at the point of ensiling. In the current study, daytime temperatures were broadly similar between the two cutting dates (14.4–19.3°C and 13.0–16.6°C, respectively). Therefore, in this instance, weather conditions are unlikely to account for the differences found in DM losses between the grass treatments over the two cuts. Although it is difficult to measure differences in DM losses during wilting that are due to plant respiration (Rotz & Muck, 1994), *F. arundinacea* var glaucescens, used in the breeding of the *Festulolium* hybrid studied here, originates from Southern France and is thereby considered “heat tolerant” (Humphreys, O’Donovan, & Sheehy‐Skeffington, 2014).

Other means by which grasses show improved water use efficiency include fewer stomata, rapid stomatal closure rate (reduced stomatal conductance) and reduced leaf surface area (Jones et al., 1981). However, *F. arundinacea* var glaucescens is amphistomatous (has equal stomata on upper and lower surface) as opposed to *Lolium* which is greater on the adaxial (upper) leaf surface, making stomatal water conductance greater in fescue than ryegrass, especially compared to lower surface of *Lolium* (Humphreys et al., 1997) but this does not explain the higher wilting rates found in ryegrass compared to the *Festulolium* in this study. More recently, crop research into resistance to abiotic stress has focussed on silica content (Zargar et al., 2019). Although previously considered a negative trait for intake of grass forage by grazing animals, silica content is now receiving new attention as a means of diversifying grasslands to combat the impact of climate change (Volaire et al., 2014). In a study by Cougnon et al. (2020) comparing silica concentrations in different forage grass species, the highest silica concentrations were found in fescue, albeit in tall fescue (*Festuca arundinacea* Schreb.). Although its silica content has not yet been reported, *F. arundinacea var glaucescens* is a progenitor of tall fescue and has contributed two of its three ancestral genomes (Humphreys et al., 1995). It may consequently also have high silica content. In contrast, amongst the grasses studied, Cougnon et al. (2020) found perennial ryegrass to contain the lowest concentrations of silica. Although at this stage speculative, possible respiration rate and silica content differences between the grasses could influence DM losses during wilting of *Festulolium* spp. when compared to ryegrasses. Future studies are now needed to understand the effects of silica content on forage wilting times as changes in current management practice of forage post‐harvest may be necessary specific to *Festulolium* to enhance its DM content in silage and to optimize its future use in conservation purposes.

#### 3.4.2 Fermentation rates

WSC provides an energy source for microbe activity during ensilage, as it does for ruminant microbes in ingested freshly grazed forage where “high sugar” ryegrasses like AberMagic have demonstrated their value as efficient livestock feed (Wilkins & Humphreys, 2003). Higher initial WSC concentration in the ryegrass compared to Lp × Fg would be expected to convey it greater advantage in terms of its potential to improve the rate of fermentation during silage production. Over the whole ensilage period, WSC concentrations were higher in Lp than Lp × Fg in silages from both cuts (*p <* 0.001) (Tables 2 and 3). This difference between the grass treatments in WSC was found despite no earlier differences in WSC at the time of cutting (Table 1). The difference in WSC between the silages was particularly evident in Cut 4, where levels in Lp were significantly higher (*p* < 0.05) at each sampling time and were found to decline slower than in Lp × Fg and with higher residual WSC after 90 days. However, overall changes in pH were similar in samples from both silages.

Although reductions in pH during the ensiling process should mitigate protein breakdown, the extent of microbial proteolytic action and plant protease activity and the relative contribution of these during the ensiling process vary over time and may still result in protein breakdown (McDonald et al., 1991; Winters et al., 2000). The optimum pH for plant proteolytic activities is pH 5–7 (Heron et al., 1989). In the current study, irrespective of harvest cut, the pH levels were still sufficiently high in the early stages of ensiling to allow plant protease activities to continue (Heron et al., 1986). Plant proteolytic activity would have been expected to decline more rapidly due to the faster decline in pH with the *Festulolium* and in the Cut 2 material. However, even at lower pH levels, microbial proteases (Filya, 2010) may continue some degree of proteolytic activity (Der Bedrosian et al., 2012; Kleinschmit & Kung Jr., 2010; Schmidt et al., 2009; Whiter & Kung, 2010).

A typical pH range for grass‐based silage is pH 4.3–4.7, although the ideal grass silage pH should range between pH 3 and 5 but will depend on the WSC and moisture content of the material ensiled (Haigh, 1990). The final pH values of all silages were within this optimal pH range (Tables 2 and 3) and indicative of a successful silage fermentation (Winters et al., 2000). Overall, from both Cuts 2 and 4, there was an interaction between grass and ensiling time (*p* < 0.001). Initial pH did not differ (*p* > 0.05) between the grasses in either cut. The decline in pH was slower in the ryegrass silages and the pH was higher (*p* < 0.05) than in the respective *Festulolium* silage in samples <1 day post‐ensiling in Cut 2 and <3 days post‐ensiling in Cut 4. Thereafter, the grasses did not differ in their pH. One explanation for the faster decline in pH in the *Festulolium* silage, compared to the ryegrass, would be their lower DM content, as research has shown that a higher DM reduces availability of metabolic water for lactic acid bacteria (Muck et al., 2018).

## 4 CONCLUSION

Increasing costs of protein feed and protein losses during ensiling are barriers to sustainable and productive ruminant systems, protein feed security and, ultimately, food security. Advances in plant breeding capable of mitigating nitrogen wastage through proteolysis either in freshly grazed or ensiled forage, and ideally both, would encourage more efficient and environmentally sustainable nutrient use by livestock and would be welcomed by the livestock industry.

In this study, the benefits of reduced proteolysis of *Festulolium* silage when conserved as ruminant feed were evidenced by lower SN concentrations in an early‐cut silage with comparable DM to ryegrass. Further research to understand the potential impact of growth ontogeny during the growing season, and the effects of respiration rates and silica content on wilting rates specific to *Festulolium* to investigate the need for change to management practice of forage post‐harvest will help to optimize use of these new grass cultivars for conservation purposes. More generally, grasses such as the *L. perenne × F. arundinacea var glaucescens* hybrid offer new opportunities to fully exploit their diverse and holistic properties (Humphreys & Zwierzykowski, 2020; Muhandiram et al., 2020). These include not only their capabilities for productive and high‐quality forage, and their efficient protein provisions but also, at a time of climate change and need for more sustainable grassland management, their multiple ecosystem service provisions.

Redesigning grasslands that can deliver high‐quality protein feed for livestock more efficiently, whilst also delivering numerous ecosystem services, is essential to sustainable global protein food security. Future work would build on the findings presented here to evaluate livestock production gains and potential environmental benefits of feeding silage made with *Festulolium* grasses.

## CONFLICT OF INTEREST STATEMENT

The authors have stated explicitly that there are no conflicts of interest in connection with this article.

## Data Availability

Data available on request from the authors.
